# Renal Function Effect on the Association Between Body Mass Index and Mortality Risk After Acute Myocardial Infarction

**DOI:** 10.3389/fcvm.2021.765153

**Published:** 2021-12-06

**Authors:** Shin Yeong Kang, Weon Kim, Jin Sug Kim, Kyung Hwan Jeong, Myung Ho Jeong, Jin Yong Hwang, Hyeon Seok Hwang

**Affiliations:** ^1^Department of Internal Medicine, Graduate School, Kyung Hee University, Seoul, South Korea; ^2^Division of Cardiology, Department of Internal Medicine, Kyung Hee University, Seoul, South Korea; ^3^Division of Nephrology, Department of Internal Medicine, Kyung Hee University, Seoul, South Korea; ^4^Department of Internal Medicine and Heart Center, Chonnam National University Hospital, Gwangju, South Korea; ^5^Department of Internal Medicine, Gyeongsang National University Hospital, Jinju, South Korea

**Keywords:** renal function, acute myocardial infarction, body mass index, mortality, cardiac death, non-cardiac death

## Abstract

**Background:** Body mass index (BMI) is a critical determinant of mortality after acute myocardial infarction (AMI), and higher BMI is associated with survival benefit in patients with renal impairment. However, there are no studies investigating the interactive effects of BMI and renal function on mortality risk after AMI occurrence.

**Methods:** We enrolled 12,647 AMI patients from Korea Acute Myocardial Infarction Registry between November 2011 and December 2015. Patients were categorized based on estimated Glomerular Filtration Rate (eGFR) and BMI. The primary endpoint was all-cause mortality after AMI treatment.

**Results:** Within each renal function category, the absolute mortality rate was decreased in patients with higher BMI. However, the adjusted hazard ratio (HR) of all-cause mortality for higher BMI was decreased as renal function worsened [adjusted HR (95% confidence interval) at BMI ≥ 25 kg/m^2^: 0.63 (0.41–0.99), 0.76 (0.59–0.97), and 0.84 (0.65–1.08) for patients with eGFR ≥ 90, 90–45, and <45 mL/min/1.73 m^2^, respectively]. There was a significant interaction between BMI and renal function (*P* for interaction = 0.010). The protective effect of higher BMI was preserved against non-cardiac death and it was also decreased with lowering eGFR in competing risks models [adjusted HR at BMI ≥25 kg/m^2^: 0.38 (0.18–0.83), 0.76 (0.59–0.97), and 0.84 (0.65–1.08) for patients with eGFR ≥ 90, 90–45, and <45 mL/min/1.73 m^2^, respectively; *P* for interaction = 0.03]. However, renal function did not significantly affect the association between BMI and risk of cardiac death (*P* for interaction = 0.20).

**Conclusions:** The effect of BMI on the mortality risk after AMI was dependent on renal function. The association between greater BMI and survival benefit was weakened as renal function was decreased. In addition, the negative effect of renal function on the BMI – mortality association was pronounced in the non-cardiac death.

## Introduction

Renal impairment is a prevalent disorder among patients with acute myocardial infarction (AMI). The incidence of renal impairment is ~30–50% in patients presenting with AMI. These patients have a high prevalence of atherosclerosis risk factors, including old age, dyslipidemia, diabetes, and hypertension (1–3). In addition, renal impairment plays a critical role in the prognosis of patients with AMI (4, 5). Previous studies have demonstrated a clear association between renal impairment and a higher risk of recurrent MI, ischemic stroke, and all-cause death after AMI treatment (2, 6). These devastating effects of renal impairment continue to increase drastically as renal function is further decreased (5, 7, 8).

Body mass index (BMI) is a parameter used to estimate cardiovascular risk in the general population, and obesity is associated with an increased risk of all-cause and cardiovascular mortality (9–11). However, if AMI occurs, the negative effect of BMI on the survival rate is paradoxically reversed. Numerous studies have reported that underweight patients have an increased mortality risk after AMI and that overweight and obese patients have better survival rates (12–15). Notably, among patients with renal impairment, a higher BMI has also been associated with mortality risk, regardless of the presence of cardiovascular comorbidities (16, 17). Studies that examined patients with renal dysfunction described paradoxically lower mortality associated with high BMI levels (16–19). These findings have led to a significant interest in how the paradoxical effect of obesity is changed when of AMI and renal impairment coexist and whether the effect of BMI on mortality is dependent on the degree of renal impairment.

Therefore, we investigated the interactive relationship between BMI, renal function, and mortality risk after AMI occurrence. We evaluated whether renal function affected the association between BMI and mortality risk after AMI and how renal function modified the effect of BMI on mortality risk.

## Materials and Methods

### Study Design and Data Source

Our data were derived from the Korea Acute Myocardial Infarction Registry (KAMIR)-National Institute of Health (NIH), which is a prospective, observational, multicenter, online registry to investigate the risk factors for mortality in patients with AMI. Patients diagnosed with ST-segment elevation myocardial infarction (STEMI) or non- STEMI (NSTEMI) from November 2011 to December 2015 were enrolled in this registry and followed up constantly. This study was conducted in compliance with the Declaration of Helsinki regarding human investigations. The study protocol was approved by the Institutional Review Board of all participating centers, and written informed consent was obtained from all patients.

We screened 13,104 patients enrolled in the KAMIR-NIH database. From this cohort, 457 patients in whom the estimated glomerular filtration rate (eGFR) and BMI could not be estimated were excluded. All patients were required to visit the outpatient department of cardiology at the end of the first month and subsequently every 3–6 month, as well as whenever angina-like symptoms occurred. Clinical follow-up was performed for 2 years after index AMI treatment. Any recorded deaths that occurred before the loss to follow-up were included in the analysis.

### Data Collection

The attending physician, with the assistance of a trained clinical research coordinator, collected data via a web-based case report system. Baseline demographics, risk factors for coronary artery disease, and laboratory data were collected at the time of admission. Data on the type of myocardial infarction (STEMI or NSTEMI) and implementation of percutaneous coronary intervention (PCI) were also collected. Left ventricular ejection fraction (LVEF) was measured using two-dimensional echocardiography. The use of certain medications was recorded during hospital stay.

### Exposure

The definition of AMI was based on the detection of an increase and/or a decrease in the levels of cardiac biomarkers (creatinine kinase-MB and troponin I or T) with at least one of the following: symptoms of ischemia, electrocardiogram (ECG) changes indicative of ischemia (ST-segment elevation or depression), development of pathological Q waves on ECG, and imaging evidence of new loss of viable myocardium or new regional wall motion abnormality (20).

Serum creatinine levels were measured at the time of hospital presentation. The eGFR was calculated using the following Chronic Kidney Disease Epidemiology Collaboration (CKD-EPI) equation (21): eGFR = 141 × min(Scr/κ,1)α × max(Scr/κ, 1) −1.209 × 0.993Age × 1.018 [if female] × 1.159 [if black], where κ is 0.7 (females) or 0.9 (males), α is −0.329 (females) or −0.411 (males), min indicates the minimum of Scr/κ or 1, and max indicates the maximum of Scr/κ or 1; eGFR= mL/min/1.73 m^2^; Scr (standardized serum creatinine) = mg/dL.

The classification of renal function was based on the Kidney Disease Improving Global Outcomes (KDIGO) guidelines for CKD stages (22, 23). Renal function was classified based on the eGFR values as follows: normal (≥90 mL/min/1.73 m^2^), mild-to-moderate impairment (90–45 mL/min/1.73 m^2^), and moderate-to-severe impairment (<45 mL/min/1.73 m^2^).

BMI was calculated as the weight in kilograms divided by the height squared in meters (kg/m^2^). Obesity status was categorized based on BMI according to the guidelines for the Asian population as follows: underweight (<18.5 kg/m^2^), ideal (18.5–23 kg/m^2^), overweight (23–25 kg/m^2^), and obese (≥25 kg/m^2^) (24). Patients were categorized into three groups according to each renal function category, and the effect of BMI on mortality rate was compared in individual renal function categories.

### Study Outcomes

The our primary endpoint was all-cause death during the 2-year follow-up after AMI treatment. The secondary endpoint was death from cardiac vs. non-cardiac causes. Cardiac death was defined as any death due to a proximate cardiac cause, such as myocardial infarction, low output failure, arrhythmia, unwitnessed death, and all procedure-related deaths, including those related to concomitant treatment (25). Non-cardiac death was caused by causes other than cardiac causes, including sepsis, multi-organ failure, and bleeding. Sudden or unexpected death was considered cardiac death, and all deaths were considered of cardiac origin unless a non-cardiac origin was documented.

### Measurement of Covariates

In the multivariable models, we used baseline data on sociodemographic variables (age, sex, and smoking), hypertension, diabetes, dyslipidemia (defined as high levels of total cholesterol, low-density lipoprotein cholesterol, and triglycerides; low levels of high-density lipoprotein cholesterol; or use of lipid-lowering medication), prevalent cardiovascular events, STEMI (defined as ST-segment elevation ≥ 2 mm in two more contiguous precordial leads or ≥ 1 mm in ≥ two or more limbs), Killip classification, LVEF, PCI, and pharmacologic therapy [aspirin, clopidogrel, β-blocker, angiotensin-converting enzyme (ACE) inhibitor or angiotensin receptor blocker (ARB), and statin]. All variables were selected based on their clinical relevance to the outcomes of interest.

### Statistical Analysis

Continuous variables, described as mean ± standard deviation and were compared using one-way ANOVA. eGFR is mean ± standard deviation and median (25th and 75th percentiles) to clarify the distribution. To compare subgroups, *post-hoc* analysis was performed using the Tukey test. Categorical data were analyzed using the Chi-square test. Cox regression was used to calculate the corresponding hazard ratios (HRs) with 95% confidence intervals (CIs) for the prognostic significance of BMI in patients with different levels of renal function. Multivariable Cox proportional hazard regression analysis determined the association of variables with MACEs after adjusting for several confounders. The multivariate models included parameters significantly associated with weight in univariate testing and clinically fundamental parameters. The interaction between eGFR and calculated BMI was assessed by entering the interaction term in a Cox proportional hazards model. We fitted competing risk regression models for each cause of death (cardiac death and non-cardiac death) to estimate the sub-distribution hazards, because cardiac and non-cardiac mortality compete with each other (26). *P*-values < 0.05 were considered as significant. All statistical analyses were performed using SPSS software (version 22.0; SPSS, IBM Corp., Armonk, NY, USA) and statistical analysis system version software (version 9.4; SAS, SAS Institute, Cary, NC, USA).

## Results

### Baseline Characteristics

A total of 12,647 patients with AMI were included. Table 1 shows the baseline demographics and clinical features according to renal function. Patients were classified into three groups according to renal function-−5,194 (41.1%) patients had normal renal function, 6,008 (47.5%) patients had mild-to-moderate renal impairment, and 1,445 (11.4%) patients with moderate-to-severe renal impairment. The mean eGFR was 102.3 ± 9.8 ml/min/1.73 m^2^, 72.2 ± 12.6 ml/min/1.73 m^2^ and 27.4 ± 12.7 ml/min/1.73 m^2^ in patients with normal renal function, mild-to-moderate renal impairment and moderate-to-severe renal impairment, respectively. The mean BMI was significantly lower in patients with moderate-to-severe renal impairment than in those with normal renal function or mild-to-moderate renal impairment. The proportion of overweight or obese patients was significantly lower among patients with moderate-to-severe renal impairment.

**Table 1:** Baseline characteristics of study population.

	**Renal function**			***P*** **-value**
	**Normal,** ***n*** **= 5,194**	**Mild-to-moderate impairment,** ***n*** **= 6,008**	**Moderate-to-severe impairment,** ***n*** **= 1,445**	
Age, (years)	56.8 ± 10.6	67.7 ± 11.6	72.7 ± 10.6	<0.001
Male, *n* (%)	4,238 (81.6)	4,334 (72.1)	829 (57.4)	<0.001
BMI, (kg/m^2^)	24.4 ± 3.3	23.9 ± 3.9	23.3 ± 3.6	<0.001
Body weight status				<0.001
Underweight, *n* (%)	134 (2.6)	264 (4.4)	112 (7.8)	
Ideal weight, *n* (%)	1,538 (29.6)	2,088 (34.8)	587 (40.6)	
Overweight, *n* (%)	1,469 (28.3)	1,580 (26.3)	334 (23.1)	
Obesity, *n* (%)	2,053 (39.5)	2,076 (34.6)	412 (28.5)	
Mean GFR, (ml/min/1.73 m^2^)	102.3 ± 9.8	72.2 ± 12.6	27.4 ± 12.7	<0.001
Median GFR, (ml/min/1.73 m^2^)	100.6 (95.1–107.1)	73.6 (62.3–83.6)	29.9 (17.0–38.4)	<0.001
SBP, (mmHg)	133.2 ± 26.4	129.2 ± 30.6	126.7 ± 34.4	<0.001
DBP, (mmHg)	81.6 ± 16.3	77.6 ± 18.3	74.2 ± 20.2	<0.001
Hypertension, *n* (%)	1,964 (37.8)	3,346 (55.7)	1,121 (77.6)	<0.001
Diabetes, *n* (%)	1,097 (21.1)	1,693 (28.2)	828 (57.3)	<0.001
Dyslipidemia, *n* (%)	591 (11.4)	696 (11.6)	152 (10.5)	0.519
History of CV event, *n* (%)	173 (3.3)	468 (7.8)	203 (14.0)	<0.001
Current Smoking, *n* (%)	2,728 (52.5)	1,977 (32.9)	280 (19.4)	<0.001
STEMI, *n* (%)	2,517 (48.5)	3,049 (50.7)	511 (35.4)	<0.001
Killip classification > I, *n* (%)	500 (9.6)	1,483 (24.7)	707 (48.9)	<0.001
LVEF, (%)	54.1 ± 10.0	51.5 ± 11.2	46.1 ± 13.2	<0.001
PCI, *n* (%)	4,750 (91.5)	5,462 (90.9)	1,167 (80.8)	<0.001
**Medical treatment**				
Aspirin, *n* (%)	5,178 (99.7)	5,983 (99.6)	1,434 (99.2)	0.059
Clopidogrel, *n* (%)	3,839 (73.9)	4,801 (79.9)	1,266 (87.6)	<0.001
β-blocker, *n* (%)	4,390 (84.5)	4,909 (81.7)	1,034 (71.6)	<0.001
ACEi or ARB, *n* (%)	4,130 (79.5)	4,761 (79.2)	910 (63.0)	<0.001
Statin, *n* (%)	4,933 (95.0)	5,485 (91.3)	1,108 (76.7)	<0.001

*Data are presented as n (%) or mean ± SD. SBP, systolic blood pressure; DBP, diastolic blood pressure; BMI, body mass index; Hb, hemoglobin; CV event, cardiovascular disease and cerebrovascular event; LVEF, center ventricular ejection fraction; ACEi, angiotensin-converting enzyme inhibitor; ARB, angiotensin receptor blocker; PCI, percutaneous coronary intervention; STEMI, ST-segment elevation myocardial infarction*.

Compared to the patients with normal renal function, those with moderate-to-severe renal impairment were older and predominantly female. The prevalence of comorbidities, such as hypertension and diabetes was significantly higher in patients with moderate-to-severe renal impairment than in those with normal or mild-to-moderate renal impairment. The proportion of STEMI was significantly lower and patients receiving PCI treatment were less prevalent among patients with moderate-to-severe renal impairment. In addition, patients with moderate-to-severe renal impairment were less likely to receive β-blockers, ACE inhibitors or ARBs, and statins.

### Mortality Rate Based on Renal Function and BMI Categories

**Figure 1:**
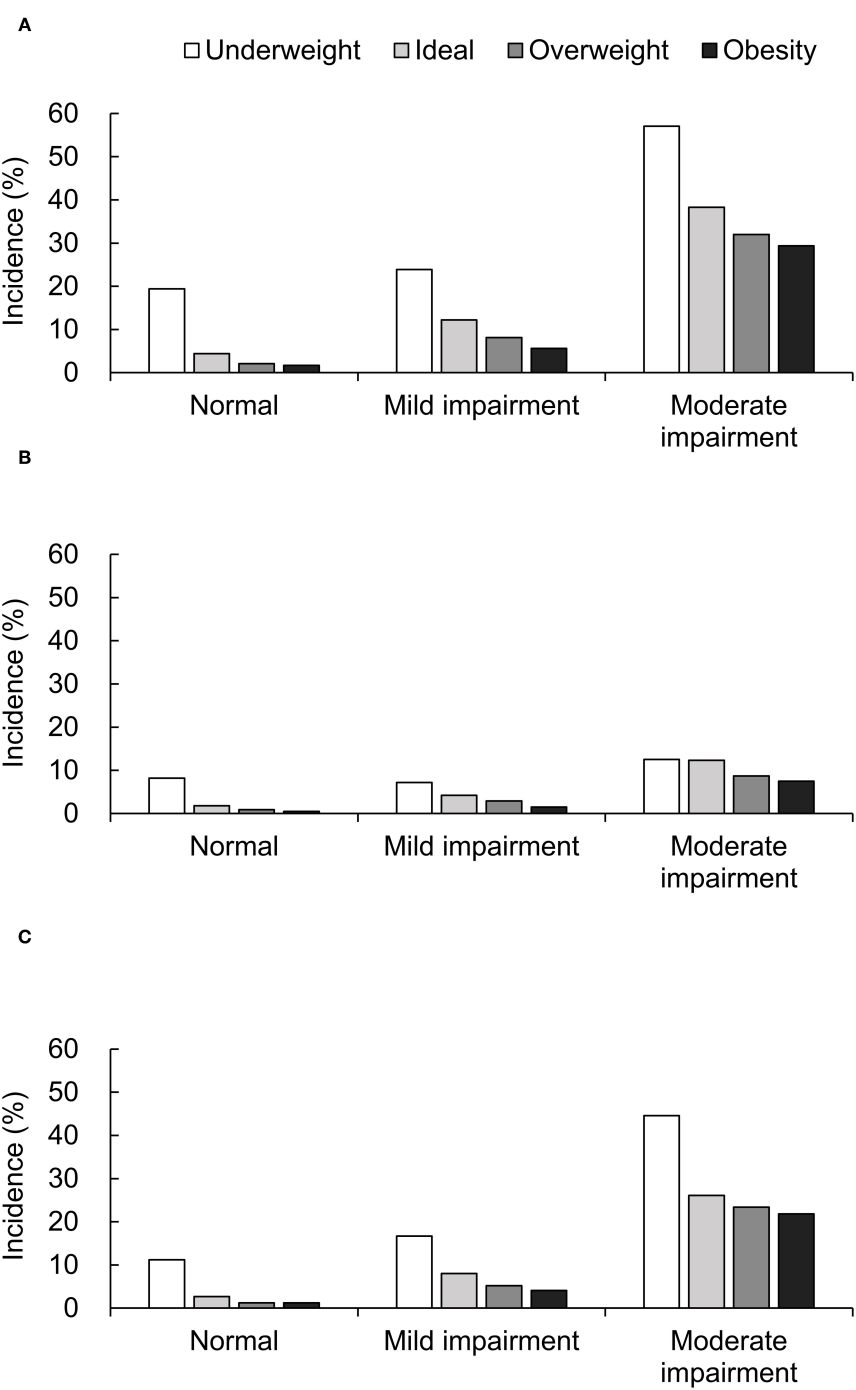
Mortality rate of all-cause **(A)**, cardiac **(B)**, and non-cardiac death **(C)** based on BMI and renal function categories.

A total of 1,238 (9.8%) deaths were observed, and the mortality rate increased with decreasing renal function (3.1, 9.4, and 35.8% for patients with normal renal function, mild-to-moderate, and moderate-to-severe renal impairment, respectively). Figure 1 shows the all-cause, cardiac and non-cardiac mortality rates based on renal function and BMI categories. Among patients with normal renal function, all-cause death (Figure 1A) was most frequently observed in underweight patients. Additionally, the mortality rates were significantly lower in overweight and obese patients than in ideal weight patients (*P* < 0.001). In patients with mild-to-moderate or moderate-to-severe renal impairment, the all-cause mortality rate decreased significantly with increasing BMI (*P* < 0.001). The rates of cardiac and non-cardiac death (Figures 1B,C) were significantly higher in underweight patients and lower in obese patients compared to ideal weight patients (all *P* < 0.001). The mortality rates of cardiac and non-cardiac causes showed similar patterns to those of all-cause death in all renal function categories.

### Renal Function and Effect of BMI on the All-Cause Mortality Risk

In the unadjusted Cox proportional model, the HR for all-cause death decreased proportionally with increasing BMI across all renal function categories (Table 2). In multivariable Cox regression analysis (Table 2), the relationship between greater BMI and reduced mortality risk remained significant for normal renal function. While a similar association between greater BMI values and survival benefit was observed in patients with mild-to-moderate renal impairment, the strength of the relationship between greater BMI and reduced mortality became weaker. The survival advantage of obesity decreases in patients with mild-to-moderate renal impairment (HR 0.76; 95% CI, 0.59–0.97), and it further decreased to insignificance in patients with moderate-to-severe renal impairment (HR 0.84; 95% CI, 0.65–1.08) compared to that in obese patients with normal renal function (HR 0.63; 95% CI, 0.41–0.99). Similarly, a higher HR for mortality in underweight patients weakened with worsening renal function [HR (95% CI): 2.18 (1.33–3.59), 1.43 (1.06–1.93), 1.33 (0.96–1.86) for normal renal function, mild-to-moderate impaired renal function, and moderate-to-severe impaired renal function, respectively]. We tested each interaction to investigate whether renal function affects the association between BMI and mortality risk. The interaction between BMI and renal function was significant in the adjusted Cox proportional model (*P* for interaction = 0.010). We repeated the analyses to demonstrate the association between BMI and all-cause mortality risk in patients with severe renal impairment (eGFR <30 ml/min/1.73 m^2^). The survival benefit of higher BMI and hazard of underweight was further reduced in this population (Supplementary Table S1).

**Table 2:** Hazard of all-cause mortality by BMI groups based on renal function categories.

	**Renal function**		
	**Normal**	**Mild-to-moderate impairment**	**Moderate-to-severe impairment**
**All cause death, unadjusted**			
Underweight	4.59^*^ (2.92, 7.21)	2.10^*^ (1.60, 2.77)	1.77^*^ (1.34, 2.33)
Ideal weight	Reference	Reference	Reference
Overweight	0.47^*^ (0.31, 0.72)	0.65^*^ (0.52, 0.80)	0.83 (0.66, 1.04)
Obesity	0.37^*^ (0.25, 0.56)	0.45^*^ (0.36, 0.56)	0.72^*^ (0.58, 0.91)
**All cause death, adjusted^a^**			
Underweight	2.18^*^ (1.33, 3.59)	1.43^*^ (1.06, 1.93)	1.33^*^ (0.96, 1.86)
Ideal weight	Reference	Reference	Reference
Overweight	0.60^*^ (0.37, 0.96)	0.92 (0.73, 1.17)	0.85 (0.65, 1.09)
Obesity	0.63^*^ (0.41, 0.99)	0.76^*^ (0.59, 0.97)	0.84 (0.65, 1.08)

*Data are presented as hazard ratios (95% confidence intervals)*.

*BMI, body mass index*.

a *The statistical model was adjusted by age, sex, Killip class, BMI, current smoker, hypertension, diabetes mellitus, dyslipidemia, previous cardiovascular disease event, previous cerebrovascular event, LVEF, type of AMI, PCI and medical treatments*.

* *P < 0.05 compared with reference BMI category*.

### Renal Function and Effect of BMI on the Risk of Cardiac and Non-cardiac Death

We analyzed the risk of cardiac and non-cardiac death in different renal function categories using a competing risk regression model (Table 3). The risk of cardiac death in the underweight patients was significantly higher in all renal function categories compared to patients with ideal weight [HR (95% CI): 2.05 (1.05–3.99), 1.61 (1.11–2.33), and 1.65 (1.12–2.42) for normal renal function, mild-to-moderate impaired renal function, and moderate-to-severe impaired renal function, respectively]. However, the effect of obesity on cardiac death was not significant across the different renal function categories. There was no significant interaction between BMI and renal function (*P* = 0.20). The risk of non-cardiac death was evaluated in the same manner. Underweight patients had a higher risk of non-cardiac death (HR 2.63; 95% CI, 1.20–5.78), and the protective effect of obesity was significant in patients with normal renal function (HR 0.38; 95% CI, 0.18–0.83). These survival benefits and disadvantages based on BMI categories diminished with worsening renal function. There was a significant interaction between BMI and renal function in the incidence of non-cardiac death (*P* for interaction = 0.03). Similar results were observed for the risk of cardiac and non-cardiac death, in patients with severe renal impairment (Supplementary Table S1).

**Table 3:** Competing risk of death from cardiac and non-cardiac cause by BMI groups based on renal function categories.

	**Renal function**		
	**Normal**	**Mild-to-moderate impairment**	**Moderate-to-severe impairment**
**Cardiac death, adjusted^a^**			
Underweight	2.05^*^ (1.05, 3.99)	1.61^*^ (1.11, 2.33)	1.65^*^ (1.12, 2.42)
Ideal weight	Reference	Reference	Reference
Overweight	0.58 (0.30, 1.11)	0.97 (0.71, 1.33)	1.03 (0.76, 1.39)
Obesity	0.87 (0.50, 1.51)	0.98 (0.71, 1.34)	1.08 (0.80, 1.45)
**Non-cardiac death, adjusted** ^**a**^			
Underweight	2.63^*^ (1.20, 5.78)	1.15 (0.68, 1.97)	0.86 (0.44, 1.67)
Ideal weight	Reference	Reference	Reference
Overweight	0.60 (0.28, 1.27)	0.88 (0.60, 1.29)	0.66 (0.42, 1.04)
Obesity	0.38^*^ (0.18, 0.83)	0.52^*^ (0.33, 0.82)	0.58^*^ (0.37, 0.92)

*Data are presented as hazard ratios (95% confidence intervals)*.

*BMI, body mass index*.

a *The statistical model was adjusted by age, sex, Killip class, BMI, current smoker, hypertension, diabetes mellitus, dyslipidemia, previous cardiovascular disease event, previous cerebrovascular event, LVEF, type of AMI, PCI and medical treatments*.

* *P < 0.05 compared with reference BMI category*.

## Discussion

BMI is an important predictor of all-cause mortality in patients after AMI; it is well-known that obesity is paradoxically associated with an improved survival rate after AMI (12, 13). Our study demonstrated that patients with AMI with normal renal function had a higher prevalence of obesity than those with impaired renal function, and the prevalence of underweight patients was higher among patients with renal impairment. These findings suggest that the distribution pattern of BMI in patients with AMI was transformed into different renal functions and that this BMI pattern is a reason for the high mortality after AMI in patients with renal impairment.

Our previous study showed that underweight patients had unfavorable clinical outcomes, including all-cause mortality after AMI (27–29). In addition, several studies consistently demonstrated that underweight was associated with higher mortality not only in patients after AMI but also in patients with impaired renal function (18, 27, 29). It has been postulated that an insufficient nutritional reserve in underweight patients renders them unstable to acute stress and unable to endure the increased metabolic demands from chronic disease conditions (30–32). Moreover, we observed that the negative effect of underweight on survival rate was consistent in all renal function categories. However, this negative effect gradually weakened as renal function worsened. We suggest that the shorter lifespan of patients with renal impairment may not allow the full presentation of the effects of underweight after AMI (33, 34).

Various have demonstrated the favorable effect of overweight and obesity on the mortality risk among patients with impaired renal function, irrespective of the history of cardiovascular events (16, 17). It also appears that the association between a higher BMI and survival benefit was strengthened as the underlying renal function declined more severely (18, 35). Therefore, considering the combined benefit of a higher BMI from AMI setting, we expected that the protective effect of higher BMI values was additionally or synergistically increased in patients with renal impairment after AMI. However, we found that the relationship between a greater BMI and survival benefit was weakened as the renal function decreased and that there was a significant interaction between BMI and the renal function. These findings indicate that the protective effect of a high BMI value affects patients with renal impairment differently from those with normal renal function. We suggest that impaired renal function and its specific conditions modify the effects of obesity on all-cause and non-cardiac mortality.

In this study, we found that obesity had no significant effect on the risk of cardiac death and that renal function did not affect the association between BMI and the risk of cardiac death. These findings are consistent with those of previous reports, showing that obesity did not increase the risk of cardiac death among patients receiving PCI and that obesity had minimal effects on cardiac death within a short-term follow up (36–38). However, these results did not extend to the long-term risk of cardiac death. Moreover, obesity increases the risk of cardiac death during long-term follow up follow-up, and the prolonged risk of cardiac death is more prominent than that of non-cardiac death (39–41). Therefore, we suggest that our study results on the association between renal function, BMI, and risk of cardiac death are valid only for short-term outcomes because the studied patients were only followed up for 2 years.

Our study showed that obesity significantly reduced the risk of non-cardiac death but not of cardiac death. In addition, the protective effect of obesity against non-cardiac death decreased with worsening renal function, and there was a significant interaction between BMI and renal function. These findings suggest that the survival benefit of a high BMI value was mainly due to the reduced risk of non-cardiac death and that renal function has greater influence on the association of a higher BMI with non-cardiac death risk. Further, patients with impaired renal function have a higher risk of non-cardiac death, and recent studies on the causes of death in patients with renal impairment predicted elevated probability of non-cardiovascular death in higher BMI categories (42–44). Therefore, it is conceivable that these adverse effects due to impaired renal function offset the survival advantage of higher BMI values, which is protective against non-cardiac causes.

There are several underlying etiologies of renal impairment and diabetic nephropathy, hypertensive nephropathy, and renal artery stenosis are largely prevalent in patients with coronary artery disease (45–47). Because renal impairment in combination with BMI is clearly associated with all-cause mortality, management of underlying nephropathy is crucial to improve patient outcomes. In cases of anatomical etiology such as renal artery stenosis, vascular intervention promotes the restoration of renal impairment. In patients with hypertensive or diabetic nephropathy, managing underlying comorbidities is a key to prevent the progression of renal dysfunction. In particular, diabetic nephropathy has known to be associated with a higher cardiovascular risk than other renal diseases, intensive management should be considered even in the case of mild renal impairment (47).

This study has some limitations. BMI may not be a reliable and accurate marker to differentiate patients with high muscle or bone mass, who are not truly overfat. Other parameters, such as waist circumference or abdominal diameter, can be added to increase the diagnostic accuracy for obesity (48, 49). In addition, information on renal function was assessed once at the time of presentation to the hospital. Therefore, we may have included individuals with acute kidney injury.

## Conclusion

The all-cause mortality rate after AMI was observed to decrease with increasing BMI in all renal function categories. However, the survival benefit of a high BMI and the hazard of a low BMI was weakened with worsening renal function, suggesting that the effect of BMI on mortality risk was dependent on renal function. In addition, the association between higher BMI benefit and renal function was more prominent in non-cardiac death risk than in cardiac death risk.

## Data Availability Statement

The raw data supporting the conclusions of this article will be made available by the authors, without undue reservation.

## Ethics Statement

The studies involving human participants were reviewed and approved by institutional review board number 05–49 of Chonnam National University Hospital. The patients/participants provided their written informed consent to participate in this study.

## Author Contributions

HH conceived the research question and designed the analysis. SK and WK drafted the manuscript and analyzed the data. JK and KJ interpreted the data. MJ and JH undertook data acquisition. All authors have read and approved the final manuscript.

## Conflict of Interest

The authors declare that the research was conducted in the absence of any commercial or financial relationships that could be construed as a potential conflict of interest.

## Publisher's Note

All claims expressed in this article are solely those of the authors and do not necessarily represent those of their affiliated organizations, or those of the publisher, the editors and the reviewers. Any product that may be evaluated in this article, or claim that may be made by its manufacturer, is not guaranteed or endorsed by the publisher.

## References

[1] Navarro MA Gosch KL Spertus JA Rumsfeld JS Ho PM. Chronic kidney disease and health status outcomes following acute myocardial infarction. J Am Heart Assoc. (2016) 5:e002772. 10.1161/JAHA.115.002772 27217497 PMC4889168

[2] Shroff GR Frederick PD Herzog CA. Renal failure and acute myocardial infarction: clinical characteristics in patients with advanced chronic kidney disease, on dialysis, and without chronic kidney disease. A collaborative project of the United States Renal Data System/National Institutes of Health and the National Registry of Myocardial Infarction. Am Heart J. (2012) 163:399–406. 10.1016/j.ahj.2011.12.002 22424010 PMC3313453

[3] Smilowitz NR Gupta N Guo Y Mauricio R Bangalore S. Management and outcomes of acute myocardial infarction in patients with chronic kidney disease. Int J Cardiol. (2017) 227:1–7. 10.1016/j.ijcard.2016.11.026 27846456

[4] Bae EH Lim SY Cho KH Choi JS Kim CS Park JW. GFR and cardiovascular outcomes after acute myocardial infarction: results from the Korea Acute Myocardial Infarction Registry. Am J Kidney Dis. (2012) 59:795–802. 10.1053/j.ajkd.2012.01.016 22445708

[5] Anavekar NS McMurray JJ Velazquez EJ Solomon SD Kober L Rouleau JL. Relation between renal dysfunction and cardiovascular outcomes after myocardial infarction. N Engl J Med. (2004) 351:1285–95. 10.1056/NEJMoa041365 15385655

[6] Lee KJ Kim SE Kim JY Kang J Kim BJ Han MK. Five-year risk of acute myocardial infarction after acute ischemic stroke in Korea. J Am Heart Assoc. (2021) 10:e018807. 10.1161/JAHA.120.018807 33372531 PMC7955456

[7] Matsushita K vanderVelde M Astor BC Woodward M Levey AS. Association of estimated glomerular filtration rate and albuminuria with all-cause and cardiovascular mortality in general population cohorts: a collaborative meta-analysis. Lancet. (2010) 375:2073–81. 10.1016/S0140-6736(10)60674-5 20483451 PMC3993088

[8] Amin AP Salisbury AC McCullough PA Gosch K Spertus JA Venkitachalam L. Trends in the incidence of acute kidney injury in patients hospitalized with acute myocardial infarction. Arch Intern Med. (2012) 172:246–53. 10.1001/archinternmed.2011.1202 22332157

[9] Calle EE Thun MJ Petrelli JM Rodriguez C Heath CW Jr. Body-mass index mortality in a prospective cohort of U.S. adults. N Engl J Med. (1999) 341:1097–105. 10.1056/NEJM199910073411501 10511607

[10] Khan SS Ning H Wilkins JT Allen N Carnethon M Berry JD. Association of body mass index with lifetime risk of cardiovascular disease and compression of morbidity. JAMA Cardiol. (2018) 3:280–7. 10.1001/jamacardio.2018.0022 29490333 PMC5875319

[11] Cercato C Fonseca FA. Cardiovascular risk and obesity. Diabetol Metab Syndr. (2019) 11:74. 10.1186/s13098-019-0468-0 31467596 PMC6712750

[12] Bucholz EM Rathore SS Reid KJ Jones PG Chan PS Rich MW. Body mass index and mortality in acute myocardial infarction patients. Am J Med. (2012) 125:796–803. 10.1016/j.amjmed.2012.01.018 22483510 PMC3408565

[13] Lamelas P Schwalm JD Quazi I Mehta S Devereaux PJ Jolly S. Effect of body mass index on clinical events after acute coronary syndromes. Am J Cardiol. (2017) 120:1453–9. 10.1016/j.amjcard.2017.07.043 28916239

[14] Romero-Corral A Montori VM Somers VK Korinek J Thomas RJ Allison TG. Association of bodyweight with total mortality and with cardiovascular events in coronary artery disease: a systematic review of cohort studies. Lancet. (2006) 368:666–78. 10.1016/S0140-6736(06)69251-9 16920472

[15] Powell BD Lennon RJ Lerman A Bell MR Berger PB Higano ST. Association of body mass index with outcome after percutaneous coronary intervention. Am J Cardiol. (2003) 91:472–6. 10.1016/S0002-9149(02)03252-6 12586271

[16] Kovesdy CP Anderson JE Kalantar-Zadeh K. Paradoxical association between body mass index and mortality in men with CKD not yet on dialysis. Am J Kidney Dis. (2007) 49:581–91. 10.1053/j.ajkd.2007.02.277 17472839

[17] Madero M Sarnak MJ Wang X Sceppa CC Greene T Beck GJ. Body mass index and mortality in CKD. Am J Kidney Dis. (2007) 50:404–11. 10.1053/j.ajkd.2007.06.004 17720519

[18] Lu JL Kalantar-Zadeh K Ma JZ Quarles LD Kovesdy CP. Association of body mass index with outcomes in patients with CKD. J Am Soc Nephrol. (2014) 25:2088–96. 10.1681/ASN.2013070754 24652789 PMC4147974

[19] Kalantar-Zadeh K Rhee CM Chou J Ahmadi SF Park J Chen JLT. The obesity paradox in kidney disease: how to reconcile it with obesity management. Kidney Int Rep. (2017) 2:271–81. 10.1016/j.ekir.2017.01.009 28439569 PMC5399774

[20] Thygesen K Alpert JS Jaffe AS Chaitman BR Bax JJ Morrow DA. Fourth universal definition of myocardial infarction. Circulation. (2018) 138:e618–51. 10.1161/CIR.0000000000000617 30571511

[21] Levey AS Stevens LA Schmid CH Zhang YL Castro AF 3rd Feldman HI. A new equation to estimate glomerular filtration rate. Ann Intern Med. (2009) 150:604–12. 10.7326/0003-4819-150-9-200905050-00006 19414839 PMC2763564

[22] KCW Group. KDIGO 2012 clinical practice guideline for the evaluation and management of chronic kidney disease. Kidney Int. (2013) (Suppl.) 2012:1–150. 10.1038/kisup.2012.72 23989362

[23] Go AS Chertow GM Fan D McCulloch CE Hsu CY. Chronic kidney disease and the risks of death, cardiovascular events, and hospitalization. N Engl J Med. (2004) 351:1296–305. 10.1056/NEJMoa041031 15385656

[24] World Health Organization. The Asia-Pacific Perspective: Redefining Obesity And Its Treatment. Sydeny: Health Communications (2000).

[25] Cutlip DE Windecker S Mehran R Boam A Cohen DJ van Es GA. Clinical end points in coronary stent trials: a case for standardized definitions. Circulation. (2007) 115:2344–51. 10.1161/CIRCULATIONAHA.106.685313 17470709

[26] Fine JP Gray RJ. A proportional hazards model for the subdistribution of a competing risk. J Am Stat Assoc. (1999) 94:496–509. 10.1080/01621459.1999.10474144

[27] Kim DW Her SH Park HW Park MW Chang K Chung WS. Association between body mass index and 1-year outcome after acute myocardial infarction. PLoS ONE. (2019) 14:e0217525. 10.1371/journal.pone.0217525 31199840 PMC6570024

[28] Bucholz EM Krumholz HA Krumholz HM. Underweight, markers of cachexia, and mortality in acute myocardial infarction: a prospective cohort study of elderly medicare beneficiaries. PLoS Med. (2016) 13:e1001998. 10.1371/journal.pmed.1001998 27093615 PMC4836735

[29] Su W Wang M Zhu J Li W Ding X Chen H. Underweight predicts greater risk of cardiac mortality post acute myocardial infarction. Int Heart J. (2020) 61:658–64. 10.1536/ihj.19-635 32641636

[30] Hogue CW Jr Stearns JD Colantuoni E Robinson KA Stierer T. The impact of obesity on outcomes after critical illness: a meta-analysis. Intens Care Med. (2009) 35:1152–70. 10.1007/s00134-009-1424-5 19189078

[31] Bucholz EM Beckman AL Krumholz HA Krumholz HM. Excess weight and life expectancy after acute myocardial infarction: the obesity paradox reexamined. Am Heart J. (2016) 172:173–81. 10.1016/j.ahj.2015.10.024 26856230 PMC5097250

[32] Oliveros H Villamor E. Obesity and mortality in critically ill adults: a systematic review and meta-analysis. Obesity. (2008) 16:515–21. 10.1038/oby.2007.102 18239602

[33] Kalantar-Zadeh K Horwich TB Oreopoulos A Kovesdy CP Younessi H Anker SD. Risk factor paradox in wasting diseases. Curr Opin Clin Nutr Metab Care. (2007) 10:433–42. 10.1097/MCO.0b013e3281a30594 17563461

[34] Turin TC Tonelli M Manns BJ Ravani P Ahmed SB Hemmelgarn BR. Chronic kidney disease and life expectancy. Nephrol Dial Transplant. (2012) 27:3182–6. 10.1093/ndt/gfs052 22442392

[35] Evangelista LS Cho WK Kim Y. Obesity and chronic kidney disease: a population-based study among South Koreans. PLoS ONE. (2018) 13:e0193559. 10.1371/journal.pone.0193559 29489920 PMC5831002

[36] Faggioni M Baber U Afshar AE Giustino G Sartori S Sorrentino S. Effects of body mass index on clinical outcomes in female patients undergoing percutaneous coronary intervention with drug-eluting stents: results from a patient-level pooled analysis of randomized controlled trials. JACC Cardiovasc Interv. (2018) 11:68–76. 10.1016/j.jcin.2017.06.060 29248412

[37] Kim BG Hong SJ Kim BK Ahn CM Shin DH Kim JS. Association between body mass index and clinical outcomes after new-generation drug-eluting stent implantation: Korean multi-center registry data. Atherosclerosis. (2018) 277:155–62. 10.1016/j.atherosclerosis.2018.08.047 30216914

[38] Choy B Hansen E Moss AJ McNitt S Zareba W Goldenberg I. Relation of body mass index to sudden cardiac death and the benefit of implantable cardioverter-defibrillator in patients with left ventricular dysfunction after healing of myocardial infarction. Am J Cardiol. (2010) 105:581–6. 10.1016/j.amjcard.2009.10.041 20185000

[39] Wolny R Maehara A Liu Y Zhang Z Mintz GS Redfors B. The obesity paradox revisited: body mass index and -long-term outcomes after PCI from a large pooled patient-level database. EuroIntervention. (2020) 15:1199–208. 10.4244/EIJ-D-19-00467 31659983

[40] Terada T Forhan M Norris CM Qiu W Padwal R Sharma AM. Differences in short- and long-term mortality associated with BMI following coronary revascularization. J Am Heart Assoc. (2017) 6:e005335. 10.1161/JAHA.116.005335 28411242 PMC5533024

[41] Nigam A Wright RS Allison TG Williams BA Kopecky SL Reeder GS. Excess weight at time of presentation of myocardial infarction is associated with lower initial mortality risks but higher long-term risks including recurrent re-infarction and cardiac death. Int J Cardiol. (2006) 110:153–9. 10.1016/j.ijcard.2005.06.040 16043245

[42] McDonald HI Thomas SL Millett ERC Nitsch D. CKD and the risk of acute, community-acquired infections among older people with diabetes mellitus: a retrospective cohort study using electronic health records. Am J Kidney Dis. (2015) 66:60–8. 10.1053/j.ajkd.2014.11.027 25641062 PMC4510204

[43] Iimuro S Kaneko T Ohashi Y Watanabe T Nitta K Akizawa T. Analysis of 2897 hospitalization events for patients with chronic kidney disease: results from CKD-JAC study. Clin Exp Nephrol. (2019) 23:956–68. 10.1007/s10157-019-01730-9 30968244 PMC6555784

[44] Navaneethan SD Schold JD Arrigain S Kirwan JP Nally JV Jr. Body mass index and causes of death in chronic kidney disease. Kidney Int. (2016) 89:675–82. 10.1016/j.kint.2015.12.002 26880461 PMC4757850

[45] Potier L Roussel R Zeller M Schiele F Puymirat E Simon T. Chronic kidney disease, diabetes, and risk of mortality after acute myocardial infarction: insight from the FAST-MI Program. Diabetes Care. (2020) 43:e43–4. 10.2337/dc19-2209 31974106

[46] Przewłocki T Kabłak-Ziembicka A Tracz W Kozanecki A Kopeć G Rubiś P. Renal artery stenosis in patients with coronary artery disease. Kardiol Pol. (2008) 66:856–62; discussion 63–4. 18803137

[47] Pálsson R Patel UD. Cardiovascular complications of diabetic kidney disease. Adv Chronic Kidney Dis. (2014) 21:273–80. 10.1053/j.ackd.2014.03.003 24780455 PMC4045477

[48] Pischon T Boeing H Hoffmann K Bergmann M Schulze MB Overvad K. General and abdominal adiposity and risk of death in Europe. New Engl J Med. (2008) 359:2105–20. 10.1056/NEJMoa0801891 19005195

[49] Ross R Neeland IJ Yamashita S Shai I Seidell J Magni P. Waist circumference as a vital sign in clinical practice: a Consensus Statement from the IAS and ICCR Working Group on Visceral Obesity. Nat Rev Endocrinol. (2020) 16:177–89. 10.1038/s41574-019-0310-7 32020062 PMC7027970

