# Supplementary material for: Renal Function Effect on the Association Between Body Mass Index and Mortality Risk After Acute Myocardial Infarction
Source: Front Cardiovasc Med. 2021 Dec 6;8:765153. doi: 10.3389/fcvm.2021.765153 (PMC8687192; doi:10.3389/fcvm.2021.765153)
Supplement: Supplementary file 1 [file Presentation_1.pptx]

## Slide 1
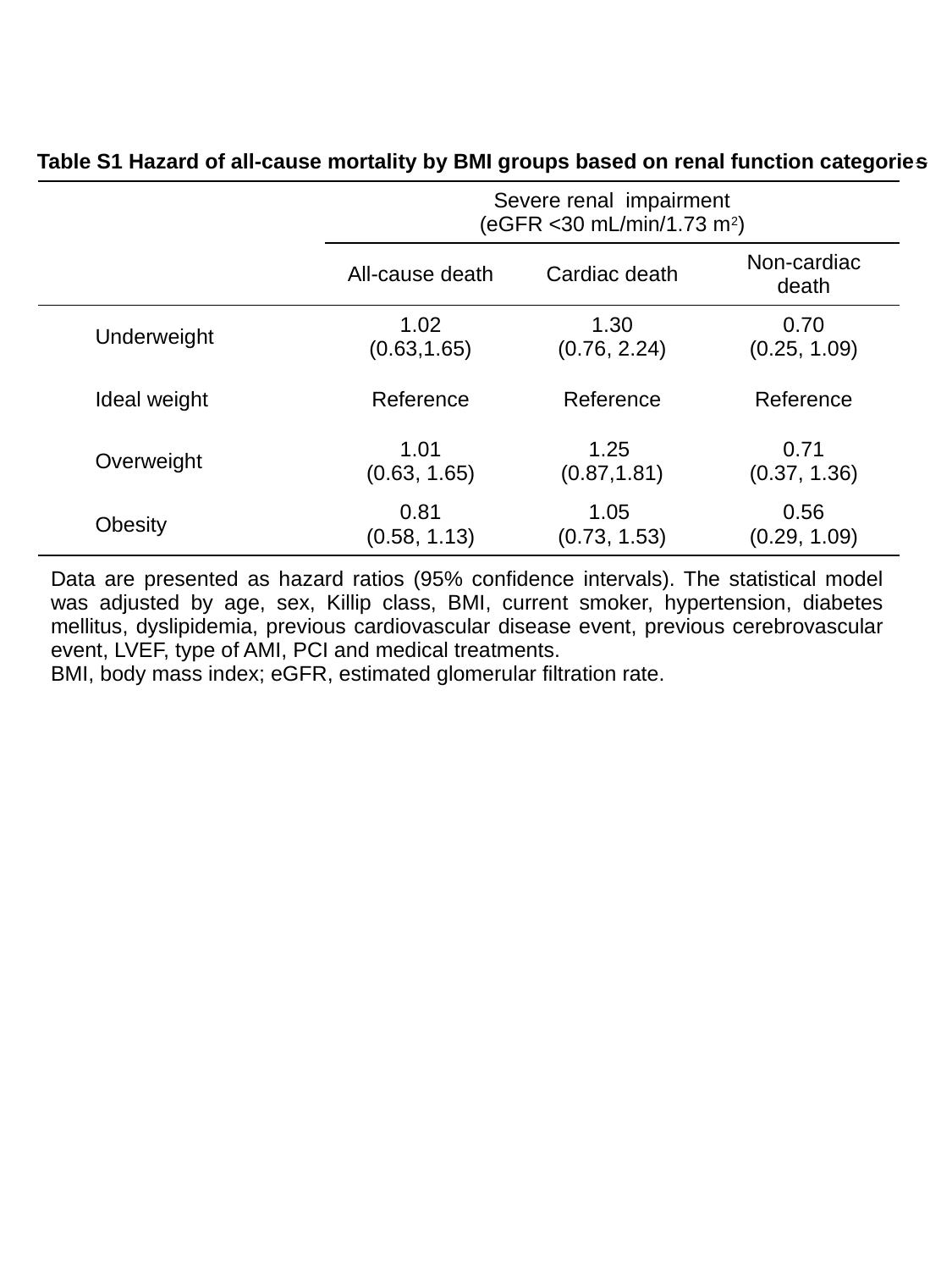

Table S1 Hazard of all-cause mortality by BMI groups based on renal function categories
| | Severe renal impairment (eGFR <30 mL/min/1.73 m2) | | |
| --- | --- | --- | --- |
| | All-cause death | Cardiac death | Non-cardiac death |
| Underweight | 1.02 (0.63,1.65) | 1.30 (0.76, 2.24) | 0.70 (0.25, 1.09) |
| Ideal weight | Reference | Reference | Reference |
| Overweight | 1.01 (0.63, 1.65) | 1.25 (0.87,1.81) | 0.71 (0.37, 1.36) |
| Obesity | 0.81 (0.58, 1.13) | 1.05 (0.73, 1.53) | 0.56 (0.29, 1.09) |
| Data are presented as hazard ratios (95% confidence intervals). The statistical model was adjusted by age, sex, Killip class, BMI, current smoker, hypertension, diabetes mellitus, dyslipidemia, previous cardiovascular disease event, previous cerebrovascular event, LVEF, type of AMI, PCI and medical treatments. BMI, body mass index; eGFR, estimated glomerular filtration rate. |
| --- |
